# The role of arbuscular mycorrhizal symbiosis in plant abiotic stress

**DOI:** 10.3389/fmicb.2023.1323881

**Published:** 2024-01-18

**Authors:** Qian Wang, Mengmeng Liu, Zhifan Wang, Junrong Li, Ke Liu, Dong Huang

**Affiliations:** ^1^Key Laboratory of Plant Resource Conservation and Germplasm Innovation in Mountainous Region (Ministry of Education), College of Life Sciences/Institute of Agro-Bioengineering, Guizhou University, Guiyang, Guizhou, China; ^2^College of Agriculture, Guizhou University, Guiyang, Guizhou, China; ^3^College of Agriculture, Guizhou Engineering Research Center for Fruit Crops, Guizhou University, Guiyang, Guizhou, China

**Keywords:** arbuscular mycorrhizal fungi (AMF), drought, salt stress, mineral nutrient, heavy metal stress, hormone

## Abstract

Arbuscular mycorrhizal fungi (AMF) can penetrate plant root cortical cells, establish a symbiosis with most land plant species, and form branched structures (known as arbuscules) for nutrient exchange. Plants have evolved a complete plant–AMF symbiosis system to sustain their growth and development under various types of abiotic stress. Here, we highlight recent studies of AM symbiosis and the regulation of symbiosis process. The roles of mycorrhizal symbiosis and host plant interactions in enhancing drought resistance, increasing mineral nutrient uptake, regulating hormone synthesis, improving salt resistance, and alleviating heavy metal stress were also discussed. Overall, studies of AM symbiosis and a variety of abiotic stresses will aid applications of AMF in sustainable agriculture and can improve plant production and environmental safety.

## 1 Introduction

According to the morphological and anatomical characteristics of mycorrhiza, the species of mycorrhiza fungi and the species of plants, mycorrhiza are usually divided into 7 types (Table 1). Arbuscular mycorrhizas (AMs) are considered to be the most familiar type of mycorrhizae in nature; they are found in approximately 72% of land plants and play important roles in the rhizosphere of plants (Genre et al., 2020). Arbuscular mycorrhizal fungi (AMF) belong to the *Glomeromycotina* in *Mucoromycota* (Bonfante and Venice, 2020). AM formation is the result of long-term co-evolution between AMF and plant roots or even leaves (Genre et al., 2020; Rich et al., 2021). The main structures of AMs include hyphae, arbuscules, and spores, and fewer species have vesicles and auxiliary cells. Mycelia can be divided into extraradical and intraradical mycelia. Extraradical mycelia are distributed in the soil, and most of them have a network structure. Extraradical mycelia can penetrate areas that cannot be reached by the plant roots and absorb water and nutrients (Gao et al., 2021; Xie K. et al., 2022). The extrinsic mycelium enters the epidermal cells of the host roots and the cortical tissue of the plants, where it forms a dendritic arbuscular structure through continuous bifurcation. The arbuscules, which are referred to as the heart of the arbuscular mycorrhizas, are considered to be the most critical structure of AMF, as they mediate the mutual exchange of many types’ nutrients between plant cells and fungi (Pimprikar and Gutjahr, 2018).

**TABLE 1:** The types, functions, and characteristics of mycorrhiza.

Types	Function	Structural diversity
Arbuscular mycorrhiza (AM)	Symbiosis with approximately 72% of land plants, which can improve the ability of hosts to resist adverse environment, promote plant growth, and also conducive to the survival of mycorrhizal fungi	Hypha entering the cells, form arbuscule, intracellular hypha circle, hypha dichotomous branching and vesicles
Ectomycorrhiza (ECM)	Produce proteases to break down proteins and provide nitrogen source for host plants	Form hypha diaphragm, mycoclena, and hartig net
Ectendomycorrhizas (EEM)	Own the dual functions of AM and ECM	Hypha entering the cells, form hypha diaphragm, mycoclena or not, hartig net, and intracellular mycelium circle
Arbutoid mycorrhizas (ARM)	Symbiosis with *Arbutus menziesii*, *Arctostaphylos uvaursi*, and *Pyrola* to improve hosts nutrition and stress resistance	Hypha entering the cells, form hypha diaphragm, mycoclena, hartig net, and intracellular hypha circle
Monotropoid mycorrhiza (MM)	Symbiosis with *Monotropa* plants and provide carbon source for host plants	Hypha entering the cells, form hypha diaphragm, mycoclena, and hartig net
Ericoid mycorrhizas (ERM)	Symbiosis with *Ericaceae* and *Epacridaceaeto*, which can promote nutrient absorption and resistance to heavy metal pollution of host plants	Hypha entering the cells, form hypha diaphragm, and intracellular hypha circle
Orchid mycorrhiza (OM)	Symbiosis with *Orchidaceae* to enhance seed germination, nutrient transport, signal transduction and stress resistance of orchids	Hypha entering the cells, form hypha diaphragm, and intracellular hypha circle

AMs play a fundamental role in ecosystems and have significant effects on plant development, water utilization, nutrition uptake, and hormone balance from biological and abiotic stresses. One of the most important ecological functions of AMs is to enhance plant biomass accumulation (Chitarra et al., 2016; Chen S. et al., 2017; Huang et al., 2020a; Zhang Q. et al., 2023). After AMF successfully invade host plant root epidermal cells to form symbionts, they can expand the effective absorption range of host plants in soil. Glycoproteins synthesized by mycelia is beneficial to the suitable rhizosphere environment creation, that enhance the host plants growth by uptake and transfer water and mineral nutrients from the outside rhizosphere (Hu et al., 2017). AMF form a direct connection between the soil and roots, which can improve plant nutrition absorption, water uptake, and photosynthetic capacity, and this reduces the negative effects of abiotic stresses such as nutrient deficiency, salt, and drought (Li et al., 2013; Liu et al., 2018; Bahadur et al., 2019; Begum et al., 2022). For example, AMF can translocate polyphosphate to the host through hyphae over a long distance by *Rhizophagus clarus aquaporin 3*, which is important for nutrient exchange between the host and fungal symbionts (Kikuchi et al., 2016). In *Medicago truncatula*, the membrane protein MtPT4 in mycorrhizal roots can also absorb the phosphate (Pi) released by AMF (Harrison et al., 2002); this protein is beneficial to plants and necessary for AM symbiosis (Javot et al., 2007). In addition to promoting plant growth and improving plant nutrient absorption capacity, AMF also plays an important role in improving plant heavy metal tolerance (Gómez-Gallego et al., 2019; Chen et al., 2023). Organic acids such as citric acid and malic acid released by AMF can be combined with metals to form complexes, and metal ions can be passivated by the chelation and filtration mechanism of extrinsic mycelium (Huang et al., 2021), so they excellent fungi for bioremediation. Table 2 depicts the studies of AM symbiosis on plant abiotic stress alleviation in different plants.

**TABLE 2:** Studies of AM symbiosis on plant abiotic stress alleviation.

Plant species	Mechanisms of influence	Effects of influence	References
*Medicago truncatula*	Increased mycorrhiza-dependent proteins synthesis	Counteracted the Cd toxicity	Aloui et al., 2009
*Lactuca sativa*	Better regulate of the ABA levels	Enhanced drought stress resistance	Aroca et al., 2008
Wheat (*Triticum aestivum* L.)	Increased N fix and auxin production	Alleviated the drought stress	Arzanesh et al., 2011
Pistachio (*Pistacia vera*)	Enhanced the uptake of PO_4_^2–^ and Zn^2+^ to maintain osmotic adjustment	Increased nutrient uptake under drought stress	Bagheri et al., 2012
Tobacco (*Nicotiana tabacum* L.)	Up-regulated the secondary metabolism, osmolyte accumulation, and antioxidant system.	Increased drought stress resistance	Begum et al., 2022
Sorghum (*Sorghum bicolor* L.)	High expression of GintAMT3	Enhanced ammonium transfer	Calabrese et al., 2016
Maize (*Zea mays* L.)	Fungal mycelium immobilization	Reduced excessive Cd transfer to the grain	Chen et al., 2004a
*Hordeum vulgare*	Increased root uranium concentrations	Reduced uranium translocation in the plant-soil continuum	Chen B. et al., 2005
Maize (*Zea mays* L.)	Increased plant growth by influencing P uptake	Alleviated Zn deficiency and contamination	Chen et al., 2004b
Cucumber (*Cucumis sativus* L.)	Upregulated RuBisCO, FBPase, F6P, Ru5PK, and related gene expression	Improved growth, nutrient uptake and photosynthesis	Chen S. et al., 2017
Black locust (*Robinia pseudoacacia* L.)	Improved photosynthesis, water status, and K^+^/Na^+^ homeostasis	Alleviated salt stress	Chen J. et al., 2017
Tomato (*Solanum lycopersicum*)	Regulated ABA and related gene expression level	Enhanced water stress tolerance	Chitarra et al., 2016
*Phoebe zhennan*	Improved photosynthesis ability	Reduced salt stress damage	Cui et al., 2021
*Arabidopsis thaliana*	Changed GA metabolism via MYB62	Changed P starvation response	Devaiah et al., 2009
*Suaeda salsa*	Changed of metabolic pathways related genes expression	Improved halophytes Na^+^ accumulation	Diao et al., 2021
*Zea mays* L.	RiHog1-RiMsn2-STREs module regulated drought stress-responsive genes in AM fungal symbiont	Improved plant drought resistance	Fan et al., 2023
*Malus robusta*	Activated hormones and Ca^2+^ signal transduction pathways	Increased calcium uptake	Fu et al., 2023
*Lotus japonicus*	LjSultr1;2 encoded a key protein involving in plant sulfate uptake	Improved plant sulfate nutritional status under S starvation	Giovannetti et al., 2014
Chicory (*Cichorium intybus* L.)	Changed expression of RiCTR1, RiCTR2 and RiCTR3A	Increased fitness of host plants under Cu deficient and toxic conditions	Gómez-Gallego et al., 2019
Maize (*Zea mays*)	Increased expression of the HMA genes and balance mineral nutrient uptake	Mitigated the changes induced by Cu toxicity	Gómez-Gallego et al., 2022
*Lotus japonicus*	Improved plant re-uptake and recycle of amino acids by LjLHT1.2	Enhanced the organic N transfer	Guether et al., 2022
*Medicago truncatula*	MtPHT2;1 influenced the phosphate transport into the chloroplast	Changed Pi transport activity	Harrison et al., 2002
*Lycium barbarum*	Maintained normal photochemical processes and higher expression levels of Rir-AQP2	Influenced water stress tolerance	Hu et al., 2017
Apple (*Malus domestica*)	Regulated genes in the MAPK pathway	Enhanced drought resistance	Huang et al., 2020a
Apple (*Malus domestica*)	MdIAA24 regulated strigolactone biosynthesis and mycorrhization	Improved drought resistance	Huang et al., 2020b
Apple (*Malus domestica*)	Regulated AM colonization by MdGH3-2/12	Reduced cadmium resistance	Huang et al., 2021
Maize (*Zea mays*)	ZmAMT3;1 transferred substantial quantities of N from AMF to plant	Enhanced the host plant’s N uptake	Hui et al., 2022
*Medicago truncatula*	MtPT4 played critical role for AM symbiosis and P transport	Enhanced P uptake	Javot et al., 2007
Soybean (*Glycine max*)	GmAMT4.1, showed specific expression in symbiosis cortical cells	Promoted active NH_4_^+^ transfer around the arbuscule branches	Kobae et al., 2010
Maize (*Zea mays*)	Increased expression of aquaporin gene GintAQPF1 and GintAQPF2	Enhanced water transport via AMF to host plants	Li et al., 2013
Maize (*Zea mays*)	IPS and 14-3GF involved in the activation of 14-3-3 protein and aquaporins	Enhanced plant drought tolerance	Li et al., 2016
*Lycium barbarum*	Enhanced IAA in leaves and roots and ABA in leaves	Increased salt stress resistance	Liu et al., 2016
Maize (*Zea mays*)	AMF and biochar had a synergistic effect on decreasing Cd phytotoxicity	Alleviated Cd stress	Liu et al., 2018
Tomato (*Solanum lycopersicum*)	Affected expression of PIPs, TIPs, and SOS genes	Enhanced salt stress tolerance	Liu et al., 2023
*Medicago truncatula*	NLP1 increased the expression of CLE35 and suppresses the CEP expression	Inhibit legume nodulation	Luo et al., 2021
Jujube (*Ziziphus jujuba*)	Balanced ion fluxes and fatty acid metabolism	Increased salt stress resistance	Ma et al., 2022
Rice (*Oryza sativa*)	Ameliorated the ΦPSII and ΦNPQ	Alleviated salt stress	Porcel et al., 2015
*Sesbania cannabina*	ABA regulated the induction of salt tolerance by SL	Enhanced salt stress resistance	Ren et al., 2018
Lettuce (*Lactuca sativa* L.) Tomato (*Solanum lycopersicum* L.)	AM symbiosis induced strigolactone biosynthesis	Improved drought tolerance	Ruiz-Lozano et al., 2016
Wheat (*Triticum aestivum* L.)	Upregulated the expression of NRT1.1, NRT2, NAR2.2, Pht1, and PT2-1	Promoted plant growth and nutrient uptake	Saia et al., 2015
*Arabidopsis thaliana*	Caused by hormonal unbalance, mainly the auxin/cytokinin ratio	Changed Cd/Cu/Zn induce root morphology	Sofo et al., 2013
*Medicago truncatula*	Strongly induced Fm201, Ri14-3-3 and RiBMH2	Improved drought or salinity stress resistance	Sun et al., 2018
Wheat (*Triticum aestivum* L.)	Increased nutrients uptake, soluble sugars, free amino acids, and proline accumulation	Mitigated the adverse effects of salinity	Talaat and Shawky, 2011
Lettuce (*Lactuca sativa* L.)	Increased soil nutrients uptake and enhance antioxidation	Promoted plant growth	Wang et al., 2024
Rice (*Oryza sativa*)	NPF4.5 played a key role in mycorrhizal NO_3_^–^ acquisition	Promoted growth and N acquisition	Wang et al., 2020
*Astragalus sinicus*	Silencing of HOG1-MAPK cascade genes led to the decreased expression of RiAQPs, RiTPSs, RiNTH1 and Ri14-3-3	Decreased drought stress resistance	Wang S. et al., 2023
*Casuarina glauca*	Enhanced ionic compartmentalization through CgNHX1, CgNHX2-1, CgCLCD, CgCLCF, and CgCLCG	Promoted salt dilution by increasing biomass and the content of K^+^	Wang S. et al., 2023
*Casuarina glauca*	Promoted growth, sustain ion balance, and increase antioxidant enzymes activity	Alleviated the adverse impact of salt stress	Wang et al., 2022a
Tomato (*Solanum lycopersicum* L.)	Promoted the expression of NADP-MEs and IAA concentration in continuous cropping substrates	Promoted plant growth and root development	Wang et al., 2021
*Lotus japonicus*	LjAMT2;2 increased the absorption of ammonium N	Promoted ammonium N transport	Wang et al., 2022b
*Medicago truncatula*	RiPT7 regulated Pi homeostasis across the fungal membrane to maintain the AM development	Promote Pi transport at the symbiotic interface	Xie X. et al., 2022
Rice (*Oryza sativa* L.)	Induced overexpression of salt tolerance related genes, improve photosynthesis and sustain ion homeostasis	Improved rice productivity in salinized soil	Zhang Y. et al., 2023
Citrus (*Citrus reticulata* Blanco)	Significantly elevated hyphal water absorption rate under drought stress	Uptake more water under drought stress	Zhang et al., 2018
*Robinia pseudoacacia* L.	Improved plant growth, regulated the concentrations and ratios of phytohormones, and increased the concentration of soil glomalin	Alleviated As toxicity	Zhang et al., 2020
*Eucalyptus grandis*	RiPho4 positively regulated the downstream components of the PHO pathway	Regulated Pi uptake and homeostasis	Zhang Q. et al., 2023
Asparagus (*Asparagus officinalis* L.)	Regulated ROS-scavenging, water and nutrient status, and cell wall synthesis and modification	Improved the salt tolerance	Zhang X. et al., 2021
*Medicago truncatula*	Decreased the NADPH oxidase-mediated H_2_O_2_ generation, and increase the antioxidant ability	Changed ROS metabolism in AM plants under Pb stress	Zhang et al., 2019

## 2 The establishment of AM symbiosis

Arbuscular mycorrhiza symbiosis relies on the formation of arbuscules for efficient nutrient exchange between plants and AM fungi. The process of symbiosis between different plants and AMFs is relatively consistent (Oldroyd, 2013). AMF need to recruit fatty acids to facilitate mycorrhizal colonization, and the *R*. *irregularis* is proved to be auxotroph of fatty acid. The transfer of fatty acids between the host plants and the fungus depends on RAM2 (REQUIRED FOR ARBUSCULAR MYCORRHIZATION 2) and the ATP-binding cassette transporter (Jiang et al., 2017). Luginbuehl et al. (2017) proved that the primary organic carbon transferred from host plants to AMF was the lipids. The transcription factor RAM1 is crucial for AM symbiosis, as it regulates the activity of glycerol-3-phosphate acyltransferase RAM2, which regulates the transfer of lipids from plants to AMF. The lipid biosynthetic enzyme FatM and ABC transporter (STR) are also required for symbiosis in the early stage and are uniquely conserved in plants engaged in AM symbiosis (Bravo et al., 2017). The transcriptional activators ERM1 and WRI5a can directly bind to the promoters then activate the expression of *FatM*, *STR*, and *STR2*; these target genes play important roles in fatty acid biosynthesis and transfer. Furthermore, the transcriptional regulatory complex ERM1/WRI5a–ERF12–TOPLESS plays an important regulatory role in arbuscule-containing cells, which help maintain a stable and beneficial symbiosis (Zhang Q. et al., 2023). In addition to obtaining fatty acids from host plants, AMF can also obtain sugars from host plants in the mature stage. The expression of Sugar Will Eventually be Exported Transporter (SWEET) family member *MtSWEET1b* is highly up-regulated in arbuscule-containing cells, and this gene has a significant effect on mediating the transport of glucose to stabilize the *Medicago truncatula* AM symbiosis (An et al., 2019).

**FIGURE 1:**
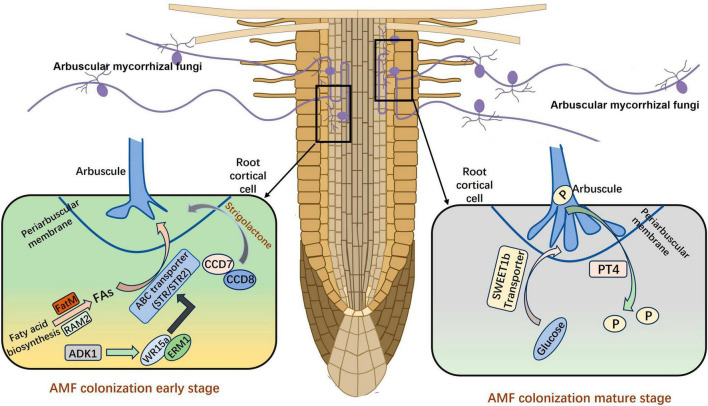
The establishment of AM symbiosis. FAs, fatty acids; P, phosphorus. The figure was created with Figdraw (https://www.figdraw.com/).

Many genes are involved in the symbiosis of AMs, in addition to genes related to fatty acid and sugar metabolism. The expression of carotenoid cleavage dioxygenase *CCD7* and *CCD8* is increased under low phosphorus (P) conditions, which facilitates the synthesis of strigolactones (SLs) and their secretion outside the root system by the transporter PDR (Kretzschmar et al., 2012). Once AMF sense the SLs secreted by plant roots, the metabolism of AMF is stimulated *in vivo*, and mycelia grow close to the root of plant which will become a host in the future (Besserer et al., 2008). Both the synthesis of SLs and the mutation of the transporter gene directly reduce the infection rate, indicating that these prophase signals are important in the establishment of symbiosis (Figure 1). Another *N*-acetylglucosamine transporter (NOPE1) has been observed in maize and rice, and previous studies have shown that AMF are unable to infect plant roots after NOPE1 mutation. This suggests that SLs may not be the only important signaling molecule in the pre-contact phase (Nadal et al., 2017). The DELLA protein binds to CCaMK and CYCLOPS to form a complex that activates the downstream GRAS transcription factor RAM1 in *Medicago sativa*, and this inhibits the arbuscular development of the *ram1* mutant (Park et al., 2015; Jin et al., 2016; Pimprikar et al., 2016). The *Arbuscule Development Kinase 1* (*OsADK1*), which is a novel kinase, is necessary for arbuscule development in rice (Guo et al., 2022). In addition, mutations of *DIPI*, *NSP1*, *NSP2*, *MIG1*, and other transcription factors can affect the development of intracellular AMs, suggesting that these transcription factors also have crucial roles in arbuscular mycorrhizal symbiosis (Maillet et al., 2011; Delaux et al., 2013; Yu et al., 2014; Heck et al., 2016).

Here, we reviewed the effects of AMF as a biological symbiont on plant growth, drought resistance, plant hormone regulation, salt tolerance, mineral nutrient absorption, and heavy metal resistance. We then focused on its potential roles in plants. Finally, future perspectives on the introduction of AM symbiosis to plants are proposed. Some of the details on plant–AMF interactions presented here have implications for research on the regulation of AM symbiosis and host plants and will aid the use of AMF in plants to enhance resistance to abiotic stress.

## 3 Regulatory roles of AMF in plants

### 3.1 AMF enhance the drought resistance of host plants

**FIGURE 2:**
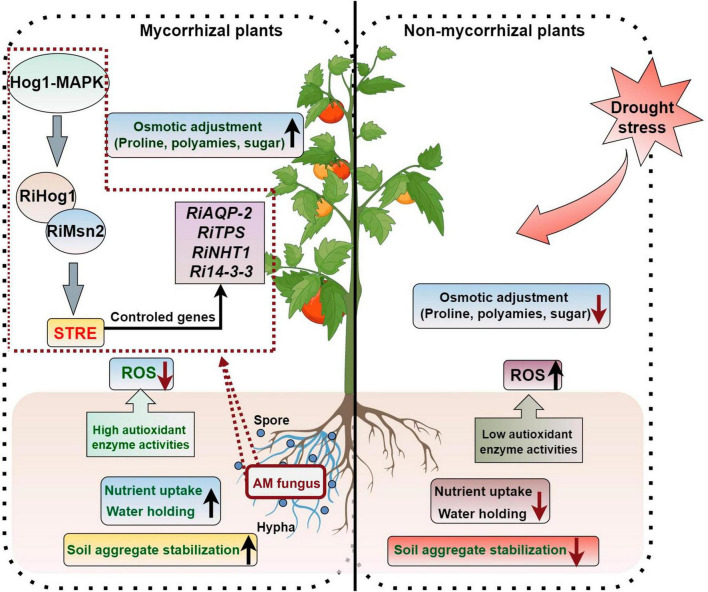
AM symbiosis regulate the response of plants to drought stress. The figure was created with Figdraw (https://www.figdraw.com/).

Drought is a mainly adverse abiotic factors stunting plant production and threatening global food security (Zhang et al., 2018). As plants are immobile and fixed organisms, drought stress can have a major effect on their biomass accumulation and development (Aroca et al., 2008). Previous studies have shown that AM symbiosis can promote the adaptation of plants to drought stress (Bahadur et al., 2019). The regulation of plant drought tolerance by AM symbiosis is a complicated process that relates to many metabolites and metabolic pathways. AMF can enhance plants drought stress resistance by (1) improving soil aggregate structures; (2) promoting the absorption of nutrient elements and water by the host plants; (3) increasing water and nutrient use efficiency of the host; (4) enhancing the osmotic adjustment ability; (5) enhancing the antioxidant capacity and alleviating the negative effects due to the accumulation of reactive oxygen species (ROS) to plants; (6) regulating hormone balances in plants; and (7) inducing the expression of stress resistance gene (Figure 2).

After the symbiotic relationship is established between AMF and host plants, AMF can expand the root volume of plants, thus increasing the area of water absorption. AMF can also improve water use efficiency and root hydraulic conductance by absorbing nutrients. Aquaporins (AQPs), which regulate water transport across the cell membrane, play an important role in plant water transport, and plasma membrane intrinsic proteins (PIPs) belong to a family of AQPs. Huang et al. (2020a) showed that the water use efficiency of *Malus hupehensis* seedlings inoculated with *R. irregularis* has a significantly higher level under drought stress than that of the non-inoculated seedlings, as the expression of *PIP1-3* and *PIP1-4* in the mycorrhizal seedling roots was significantly up-regulated compared with the untreated plants, and the expression level of *Rir-AQP1* and *Rir-AQP2* in the AMF was up-regulated under drought. Li et al. (2013) found that the AQP genes *GintAQPF1* and *GintAQPF2* was significantly up-regulated in extra-root hyphae and infected mycorrhizae under drought stress, which suggests that AMF have directly involvement in the response process to drought stress of plants. D-myo-inositol-3-phosphate synthase (IPS) and 14-3-3-like protein GF14 (14-3GF) are responsible for the signal communication between maize and AMF under drought stress condition, and co-expression of these two genes can enhance maize drought tolerance (Li et al., 2016).

The transcription factor RiMsn2 from *R. irregularis* is essential for arbuscule formation and can enhance the drought tolerance of plants. RiMsn2 can interact with RiHog1 and regulate the STRE-controlled genes from *R. irregularis*; the RiHog1-RiMsn2-STREs module regulates the drought stress response genes expression of AM fungal symbionts (Fan et al., 2023). Three Hog1 (High Osmolarity Glycerol 1)-MAPK cascade genes from *R. irregularis*, named *RiSte11*, *RiPbs2*, and *RiHog1* have been identified and the silencing of those genes resulted in the reduced expression of drought-resistance genes (*RiAQPs*, *RiTPSs*, *RiNTH1*, and *Ri14-3-3*), which inhibited the development of arbuscule and reduced the resistance ability of host plants to drought stress (Wang S. et al., 2023). The encoding 14-3-3-like protein genes *Ri14-3-3*, *Fm*2*01*, and *RiBMH2* have been shown to participate in arbuscule formation and make responses to drought stress in host plants (Sun et al., 2018).

### 3.2 AMF improve the mineral nutrient absorption of host plants

Mineral nutrient has an important effect on plant abiotic stress resistance, such as drought stress (Shi et al., 2017). Wang et al. (2019) reported an enhancement of antioxidant enzyme activities under normal nitrogen (N) supply conditions compared with a low N treatment, which effectively inhibited the accumulation of ROS and consequently reduced the damage caused by drought stress on plants. After forming a symbiotic relationship with host plants, AMs can promote the absorption of a large number of nutrients, usually P and N (Fu et al., 2023; Shi et al., 2023). This stems from the extra-root hyphae of AMF, which are 1–2 times smaller in diameter than plant roots. These hyphae can thus penetrate deeper into the soil to absorb nutrients and expand the absorption area, which can promote root growth and increase nutrient uptake (Smith et al., 2010). The most important effect of AMF is considered to be promote the uptake of P by host plants (Wang et al., 2024). The *R. irregularis* Pi transporter RiPT7, containing an SPX (SYG1/Pho81/XPR1) domain, contributes to the inflow and outflow of Pi across to the plasma membrane according to the Pi gradient. *RiPT7* silencing impedes *R. irregularis* mycorrhizal symbiosis and Pi delivery under low-Pi conditions (Xie X. et al., 2022). Shi et al. (2021) proved that the P starvation response (PHR) transcription factors are needed for mycorrhizal symbiosis and the PHR2 can regulate the expression of mycorrhizal symbiosis-related genes by the P1BS motif. Another *R. irregularis* transcription factor RiPho4, which contains an HLH domain and is significantly induced by P starvation, play a positive role on the downstream phosphate (PHO) pathway genes *RiPT1*, *RiPT2*, and *RiPT3* in *R. irregularis* (Zhang S. et al., 2023). The GigmPT, a high-affinity Pi transceptor in *Gigaspora margarita*, is necessary for AM symbiosis development, which can activate both the Pi signaling pathway and the signaling cascade of protein kinase A (Xie et al., 2016). Kumar et al. (2008) found that P in soil forms a complex with calcium (Ca) and magnesium (Mg), which is usually not conducive to plant absorption. However, high acid phosphatase activity stemming from AMF can promote the release of P from these complexes.

**FIGURE 3:**
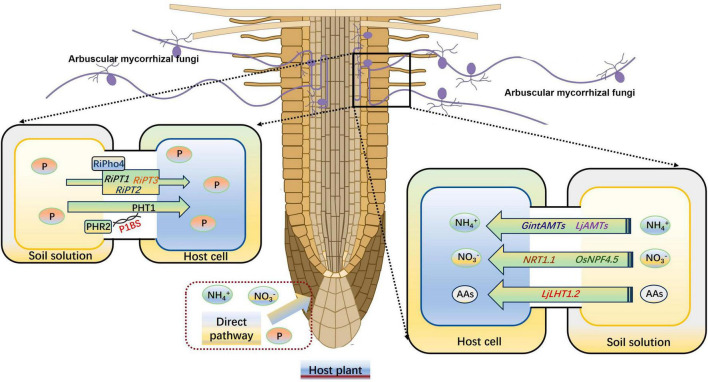
AM symbiosis enhances the uptake of P and N by plants. N, nitrogen; P, phosphorus. AAs, amino acids. The figure was created with Figdraw (https://www.figdraw.com/).

Arbuscular mycorrhizas can effectively increase the N uptake and utilization by host plants, as they not only transfer N between fungi and host plants but also between different plants by hyphal bridges (Gao et al., 2021). AMF has its own N transport system, and three AMT genes (*GintAMT1*, *GintAMT2*, and *GintAMT3*) have been identified from *R. irregularis* (Kobae et al., 2010). *GintAMT1* and *GintAMT2* are expressed in both extraneous and intraradicular mycelium, and they are involved in NH_4_^+^ absorption of AMF and recovery of NH_4_^+^ loss at the symbiotic interface due to fungal metabolism (Pérez-Tienda et al., 2012). *GintAMT3* is highly induced only in intraradicular mycelium, and its expression is regulated by the substrate concentration and carbon source (Calabrese et al., 2016). Symbiotic plant N uptake from the peri-arbuscular space depends on plant N transporters that localized in the peri-arbuscular membrane, such as the AMF induced NO_3_^–^ transporter OsNPF4.5 in *O. sativa*, the amino acid transporter LjLHT1.2, and the NH_4_^+^ transporter LjAMT2;2 in *Lotus japonicus* roots (Wang et al., 2020, 2022b; Guether et al., 2022; Zhang Y. et al., 2023). In maize (*Zea mays*) and sorghum (*Sorghum bicolor*), the expression level of NPF4.5 homologs also up-regulated by the AMF infection, indicating that the NO_3_^–^ absorption pathways are highly active under AM symbiosis (Wang et al., 2020). Saia et al. (2015) indicated that AM symbiosis significantly increase the expression level of *NRT1.1*, *AMT1;2*, and *AMT2;1* in durum wheat (*Triticum durum* Desf.). Another AMF-inducible ammonium transporter *ZmAMT3;1*, which specifically expressed in cortical cells that were AMF infected, can absorb 68–74% of the N transported from AMF to plants in maize (Hui et al., 2022; Figure 3). In *Medicago truncatula*, two signaling peptides, C-terminally encoded peptides (CEPs) and CLAVATA3/endosperm surrounding region-related peptides (CLEs), positively and negatively regulate symbiotic nodule development, respectively, in response to NO_3_^–^ levels (Oldroyd and Leyser, 2020; Luo et al., 2021). Additionally, AM colonization can enhance plant potassium, sulfur, zinc (Zn), iron, Mg, and Ca uptake in various plants (Talaat and Shawky, 2011; Garcia and Zimmermann, 2014; Giovannetti et al., 2014).

### 3.3 AMF enhance the salt tolerance of host plants

Salt stress affects plants growth in many regions around the world, especially irrigated land, and limits the healthy development of agricultural industries and the distribution of plants in the world (Cui et al., 2021). Approximately one in five of irrigated land in the world is influenced by salinization, and even worse, this figure is continually expanding. The adverse effects of salt stress on plants mainly stems from the decrease of available water as Na^+^ accumulates around the roots and the toxic actions of Na^+^ and Cl^–^ on plants, which disrupt the life processes of plants (Parihar et al., 2015). Plants have developed a series of adaptive strategies to cope with salt stress that relate to signal transduction such as calcium signal, phosphatidylinositol, protein kinases, and phytohormones. These lead to adaptive responses, such as compatible solute production, ion efflux and segregation, ROS homeostasis regulation, and the growth rate change. In addition, plants can establish symbiotic relationships with beneficial rhizosphere microorganisms to deal with the adverse effects of salt stress, such as AMF (Porcel et al., 2015; Evelin et al., 2019).

Zhang X. et al. (2021) conducted transcriptome sequencing on asparagus (*Asparagus officinalis* L.) seedling roots and found that 391 differentially expressed transcripts under salt stress were regulated by AMF, and these were mainly involved in ROS eliminating, mineral elements and water uptake regulation, and cell wall functional construction. Under salt stress conditions, seedlings of mycorrhizal halophytes have lower sodium ion concentrations and lower soluble sugar concentrations than non-mycorrhizal halophytes. These differences might be related to the carbohydrate and energy metabolism regulation, for example the glyoxylate and dicarboxylic acid metabolic pathways (Diao et al., 2021). Cui et al. (2021) proved that AMF improved the chlorophyll content of leaves, photosynthetic rate, and fluorescence correlation parameters and enhanced the utilization of light energy of *Phoebe zhennan*, which alleviated the damage induced by salt and promoted the growth of *P. zhennan* in saline–alkali soil. In addition, the Na^+^/K^+^ ratio is changed in AM symbiosis plants to sustain the osmotic equilibrium state when subjected to salt stress (Hdar et al., 2023). AMF colonization can enhance jujube root H^+^ efflux and K^+^ influx and fatty acid metabolism during salt stress, and the fatty acid content is increased in leaves and roots of AM symbiosis plants, which confers greater salt stress in wild jujube. The plasma membrane ATPase gene *ZjAHA7* is activated by mycorrhizal colonization, which initiates H^+^ efflux; the expression of *ZjHAK2* is also up-regulated, and this promotes K^+^ accumulation in AM plants to achieve a high K^+^/Na^+^ state (Ma et al., 2022).

**FIGURE 4:**
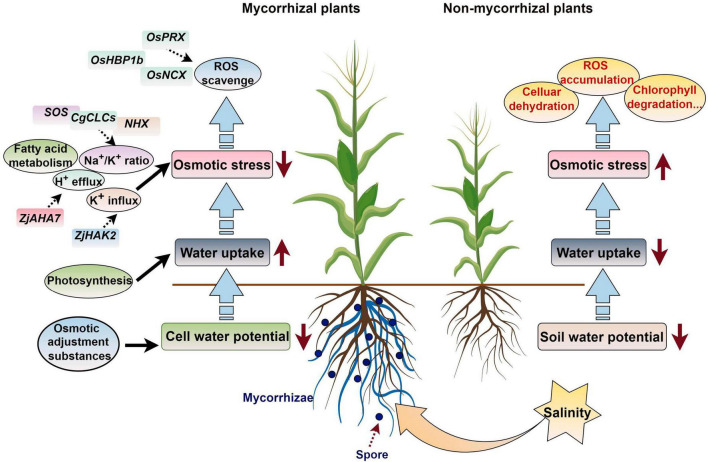
Working model of how AM symbiosis regulates the response of plants to salt stress. ROS, reactive oxygen species. The figure was created with Figdraw (https://www.figdraw.com/).

When subjected to salt stress, the expression of the salt tolerance-related genes *OsPRX*, *Os10g*, *OsHBP1b*, and *OsNCX* in mycorrhizal plants was higher than that in non-mycorrhizal plants, which increased the ROS scavenging ability and decreased malondialdehyde accumulation in rice (Zhang B. et al., 2023). Wang et al. (2022a) found that the expression of some ion transfer, ROS scavenging, and carbohydrate metabolism related genes, including HAK5, PIP1-2, MYB46, NAC43, GLP10, SKOR, CPER, and WRKY19 was significantly enhanced by *R. irregularis*, which increased the salt stress tolerance of *C. glauca*. When AMF is applied to *Casuarina glauca*, the expression of some *CgNHXs* and *CgCLCs* can increase plant biomass, the K^+^ content, and the compartmentalization of Na^+^ and Cl^–^ in vacuoles, which improves salt tolerance (Wang Y. et al., 2023). After AM symbiosis, the up-regulation of *SOS1/NHX7* in *Robinia pseudoacacia* roots can enhance saline stress tolerance by promoting the efflux of Na^+^ from plant roots (Chen J. et al., 2017). The salt overly sensitive (SOS) genes *SlSOS1* and *SlSOS2* is up-regulated by AMF colonization when subjected to salt stress, which enhances the salt tolerance of tomatoes (Liu et al., 2023). Ren et al. (2018) found that the hub genes in the module related to the response to salt stress in *Sesbania cannabina* inoculated with AMF included a range of transcription factors, such as WRKY, ERF, MYB, and TCP members. These results provide new bases for further understanding the regulatory mechanisms underlying plant–AMF interactions during salt stress responses (Figure 4).

### 3.4 AM symbiotic regulation of plant hormones to alleviate abiotic stress

Plant hormones are multifunctional substances that play key roles in plant life cycle, including the plant–AMF interactions process (Foo et al., 2013; Charpentier et al., 2014; Etemadi et al., 2014). Auxin (IAA) is a key endogenous plant hormone that has been identified to play a very important role in arbuscular development process in plants (Etemadi et al., 2014; Wang et al., 2021). Meixner et al. (2005) proved that soybean roots inoculated with AMF had significantly higher IAA concentrations than those in the uninoculated control. AMF can promote the development of roots, with emphasis on the lateral roots, by increasing indole-3-acetic acid (IAA) levels (Arzanesh et al., 2011). Ma et al. (2022) showed that AM symbiosis alters phytohormonal levels and that the IAA and abscisic acid (ABA) content is significantly increased in jujube roots after mycorrhizal formation but reduced by salt stress.

Strigolactones are important for symbiotic fungi, especially AMF (Mitra et al., 2021). Drought stress decreases SL content in both non-AMF-colonized and AMF-colonized plants (Ruiz-Lozano et al., 2016). Huang et al. (2020b) showed that overexpression of the auxin/indole-3-acetic acid gene *MdIAA24* can promote the mycorrhizal infection of apple roots by increasing the content of SLs, which results in a higher mycorrhizal infection rate and greater drought resistance of transgenic lines. The SL contents are negative correlation with plant P status and directly regulation the process of AM symbiosis (Yoneyama et al., 2007). Low P in plants induces the genes expression relating to SL biosynthetic, and two GRAS transcription factors (NSP1 and NSP2) are responsible for mediating this response; these two transcription factors also take part in the AM symbiosis signaling pathway (Liu et al., 2011; Luginbuehl and Oldroyd, 2017). Gibberellic acid (GA) is another important hormone involved in regulating changes associated with plant P status and AM symbiosis. The biosynthesis of GA is regulated by P status in plants, and low P stress down-regulates the expression of GA biosynthesis related genes but enhances the transcription of *DELLA* genes (Devaiah et al., 2009). Floss et al. (2013) showed that the arbuscule development process is affected by DELLA proteins, which act as repressors of GA signaling and stunt plants biomass accumulation and development process. Under AM symbiosis, the expression levels of genes related to the degradation and signaling of GA are altered substantially (Yu et al., 2014).

**FIGURE 5:**
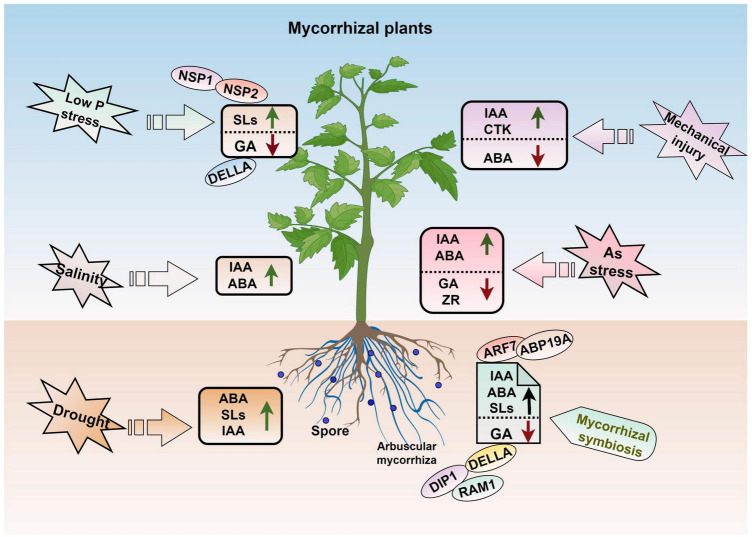
AM symbiosis and hormone regulation in plant abiotic stress. IAA, auxin; ABA, abscisic acid; GA, gibberellic acid; SLs, strigolactone; ZR, zeatin riboside; CTK, cytokinin. The figure was created with Figdraw (https://www.figdraw.com/).

Arbuscular mycorrhizal fungi play an important role in maintaining hormone balances in plants (Figure 5). Zhang J. et al. (2021) found that AMF maintain hormone balances after roots are damaged by inducing significant increases of IAA and cytokinin levels but decreases in ABA levels in both the roots and leaves, which leads to increases in the ratio of fine roots and promotes root growth. In addition, AMF can respond to drought stress by triggering plant hormone signaling. Increased ABA biosynthesis ability in both AM mycorrhizal and stressed plants can increase the establishment of AM symbiosis and improve drought tolerance (Bahadur et al., 2019). Co-inoculation with AMF and rhizobacteria can improve the ABA and IAA contents in the tobacco shoots when subjected to drought stress (Begum et al., 2022). Inoculation of watermelon with different numbers of AMF spores revealed that the concentrations of ABA, IAA, GA3, and zeatin riboside (ZR) were differentially altered as the number of spores increased. For example, ABA concentrations were highest when 300 spores were inoculated per plant and lower when 600 spores were inoculated per plant (Wu et al., 2021). In *Lycium barbarum* L. (Goji), the IAA content in both the leaves and roots and ABA content in the leaves are enhanced after inoculation with AMF, and this maintains the osmotic balance and improves salt stress resistance (Liu et al., 2016). Under arsenic (As) stress conditions, AMF-inoculated plants can regulate the concentrations and ratios of phytohormones to alleviate stress, which includes an increasing in IAA and ABA contents but a decreasing in the GA and zeatin riboside contents (Zhang et al., 2020).

### 3.5 AMF symbiosis enhances heavy metal resistance in plants

Heavy metal pollution such as cadmium (Cd), mercury (Ag), As, and lead (Pb) has become increasingly severe due to the excessive use of pesticides in agricultural production, large-scale livestock and poultry farming, and industrial discharge (Wu et al., 2018). Heavy metals, take Cd stress as an example, generally cause damage to plants in two biological pathways. First, when Cd are absorbed by plant roots, they disturb the ion balance within the plant, impede normal life activities, and induce metabolic activity disorders. Second, after Cd is absorbed by the plant, it may form complexes with proteins, enzymes, and other organic biological macromolecules to take the place of the necessary elements in the original structure of enzymes and other biological macromolecules, which makes them lose specific functions, become inactivated, and even denatured. Usually, plants undergo various physiological and molecular changes to escape or mitigate the protein inactivation and oxidative damage due to the ROS burst when exposed to Cd stress (Sofo et al., 2013). AM symbioses have been proved to enhance the resistance of plants to soil contaminated with heavy metals, such as Cd (Chen et al., 2004a), Pb (Chen X. et al., 2005), copper (Cu) (Chen et al., 2007), Zn (Chen et al., 2004b), and uranium (Chen X. et al., 2005).

Two common mechanisms have been shown to improve the heavy metal resistance of plants through mycorrhizal symbiosis: the “growth dilution effect” and “mycorrhizal immobilization.” That is, AM symbiosis can increase plant P content, which promotes the biomass accumulation of plants and the dilution of heavy metals in plants, or AMF can restrict heavy metals within the plant roots by precipitating polyphosphate complexes, thereby inhibiting their transfer to the shoots (Wu et al., 2015; Liu et al., 2018). Chen X. et al. (2005) suggest that mycorrhizae under Pb stress can facilitate plant growth via improving P absorb and mitigating Pb stress toxicity through fixing more Pb in the roots. AMF can also colonize plants in natural As-contaminated soils and alleviate As phytotoxicity (Sun et al., 2016). In addition, AM symbiosis can contribute to methylation (Li et al., 2018), which provides a new pathway by which AMF can alleviate heavy metal phytotoxicity. AMF can also enhance the antioxidant enzyme activity of plants by secreting lipids and proteins or changing the expression level of heavy metal stress respond related genes, thereby reducing the serious damage of heavy metal stress to plants (Aloui et al., 2009). Zhang et al. (2019) showed that AM plants might sustain ROS homeostasis under exposure to Pb stress by down-regulating the expression of the respiratory burst oxidase homologs (RBOHs) gene *MtRbohC-G*, decreasing the H_2_O_2_ accumulation in *Medicago truncatula*. When subjected to heavy metal stress, AMF can induce the hormone synthesis, including IAA, ABA, and GA, in host plants so as to enhance resistance to heavy metal stress. Under Cd stress, AM symbiosis results in an increase in ABA in a high Cd uptake maize cultivar and an increase in IAA in a low Cd uptake maize cultivar (Chen et al., 2023).

Some related genes in plants also take part in the AMF-mediated responses process to heavy metal stress. In maize, the expression of heavy metal ATPase (HMA) ZmHMA3a and ZmHMA4 isoforms was induced by Cu treatment in mycorrhizal plants, suggesting that AM symbiosis enhanced the expression of *HMA* genes putatively encoding Cu detoxification proteins to alleviate Cu toxicity by AM (Gómez-Gallego et al., 2022). Gómez-Gallego et al. (2019) identified two Cu transporter (CTR) family members (*RiCTR1* and *RiCTR2*) and a CTR-like protein (RiCTR3A) from *R*. *irregularis*; these three genes are involved in Cu transport and tolerance. Further analysis showed that RiCTR3A may act as a Cu receptor under Cu stress. Three other genes (*RiATOX1*, *RiSco1*, and *RiSSC*) from *R. irregularis* have been identified, and these genes encode putative chaperones that separately mediate the transfer of Cu to ATPases, cytochrome C oxidases, and Cu or Zn superoxide dismutase (Ferrol et al., 2016). Huang et al. (2021) showed that AMF inoculation can reduce Cd accumulation in apple, and the *MdGH3-2/12* silenced seedlings appeared lower Cd stress resistance for which reduced the AM symbiosis ratio compared to wild type.

## 4 Conclusion and future perspectives

**FIGURE 6:**
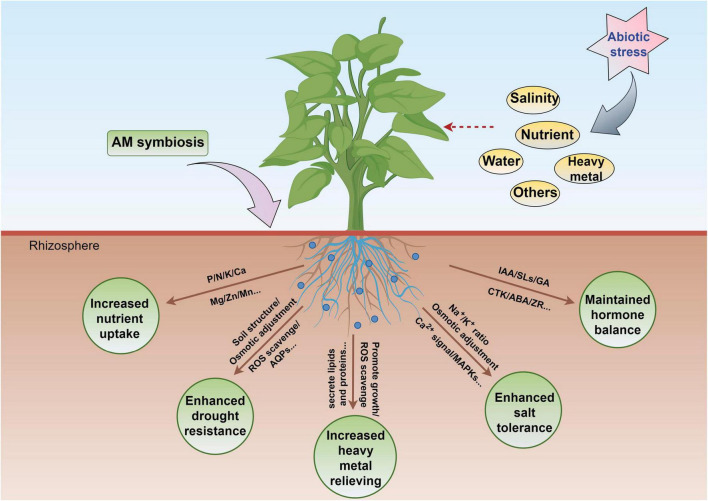
Some potential applications of AMF in plants exposed to abiotic stress. The figure was created with Figdraw (https://www.figdraw.com/).

Arbuscular mycorrhizal fungi assume a crucial role in the plant life cycle, and the generation of AM is regarded as the final results of long-term co-evolution between plants and AMF. Its functions include (1) increasing the absorption of P and other nutrient elements for example Ca, Zn, Cu, and N by host plants; (2) improving the resistance of hosts to drought, cold, and saline–alkali stress, as well as various diseases, including soil-borne diseases; and (3) promoting the generation of soil aggregate’s structure and improving the ability of soil to retain water and fertilizer. The present review mainly highlighted the effects of interactions between AMF and host plants on drought resistance, nutrient uptake, hormone regulation, salt resistance, and heavy metal stress resistance (Figure 6).

Though AM fungi has great potential applications in plant production, the successful application of AM fungi in agriculture still has severe key microbial technical challenges. First, it is difficult to produce the inoculum on a large scale, on account of the nature of obligate biotrophy of AMF. Some pure culture approaches have recently been born (Kameoka et al., 2019; Sugiura et al., 2020; Tanaka et al., 2022), which will bring hope to obtain high yield and high-quality AM fungal by asymbiotic mass production. Second, different strains should be selected in different crop systems based on plant and AM fungal genotypes, colonization ability and symbiotic efficiency. Moreover, it urgently needs to establish a quality management framework for AM fungal inoculants in agricultural practices to further optimize the purity and infection rate of AMF inoculants that used in production (Salomon et al., 2022). Strengthening the research on the functional diversity and purification of AMF will help the development of AMF related industries, promote the wide application of AMF in agricultural production, and provide important support for sustainable agricultural development.

## Author contributions

QW: Funding acquisition, Investigation, Writing—original draft. DH: Funding acquisition, Supervision, Writing—review and editing. ML: Investigation, Writing—original draft. ZW: Investigation, Writing—original draft. JL: Data curation, Investigation, Writing—original draft. KL: Investigation, Writing—original draft.
